# Targeted dual-receptor phage cocktail against *Cronobacter sakazakii*: insights into phage-host interactions and resistance mechanisms

**DOI:** 10.3389/fmicb.2024.1468686

**Published:** 2024-12-06

**Authors:** Seongok Kim, Bokyung Son, Yeran Kim, Hyeongsoon Kim, Gahyeon Nam, Hakdong Shin, Sangryeol Ryu

**Affiliations:** ^1^Department of Food Science and Biotechnology, Carbohydrate Bioproduct Research Center, College of Life Science, Sejong University, Seoul, Republic of Korea; ^2^Department of Food Biotechnology, Dong-A University, Busan, Republic of Korea; ^3^Department of Food and Animal Biotechnology, Research Institute of Agriculture and Life Sciences, Seoul National University, Seoul, Republic of Korea; ^4^Department of Agricultural Biotechnology, Seoul National University, Seoul, Republic of Korea; ^5^Research Institute of Agriculture and Life Sciences, Seoul National University, Seoul, Republic of Korea; ^6^Center for Food and Bioconvergence, Seoul National University, Seoul, Republic of Korea

**Keywords:** *Cronobacter sakazakii*, phage cocktail, flagella, lipopolysaccharides, bacteriophage therapy, phage resistance, fitness trade-off

## Abstract

**Introduction:**

*Cronobacter sakazakii* is a notorious foodborne pathogen, frequently contaminating powdered infant formula and causing life-threatening diseases in infants. The escalating emergence of antibiotics-resistant mutants has led to increased interest in using bacteriophage as an alternative antimicrobial agent.

**Methods:**

Two phages, CR8 and S13, were isolated from feces and soil samples and their morphology, physiology, and genomics were characterized. Phage receptor was determined using deletion mutants lacking *flgK, rfaC, fhuA, btuB, lamb*, or *ompC* genes, followed by complementation. Phage-resistant mutants were analyzed for phenotypic changes and fitness trade-offs using motility assays and Caco-2 cell invasion models.

**Results:**

CR8 and S13 were identified as members of *Caudoviricetes*. Phage CR8 and phage S13 utilize flagella and LPS, respectively, to adhere to host cells. Bacterial challenge assay demonstrated delayed emergence of the resistant mutant as well as stronger lytic activity of a phage cocktail consisting of CR8 and S13 than the single phage treatment. Phenotypic analysis of the phage cocktail resistant strain, designated as CSR strain, revealed that the resistance resulted from the impaired receptor proteins for phage, such as defects in motility and alteration in LPS structure. CSR strain exhibited significant attenuation in invading human intestinal epithelial Caco-2 cells compared to WT cells.

**Conclusion:**

This study demonstrates that the development of the phage cocktail targeting distinct host receptors can serve as a promising antimicrobial strategy to effectively control *C. sakazakii*.

## 1 Introduction

*Cronobacter sakazakii*, a gram-negative food-borne pathogen, has been classified by International Commission on Microbiological Specification for Foods as a “Severe hazard for restricted populations, causing life threatening or substantial chronic sequelae or long duration” (Tompkin, 2002). *C. sakazakii* is particularly notorious to neonates due to its ability to resist desiccation, which allows it to survive in dry conditions such as powdered infant formula. Infection caused by *C. sakazakii* can lead to necrotizing enterocolitis, septicemia, and meningitis in infants and immunocompromised individuals with a mortality rate up to 55% and even survivors often suffer from significant neurological sequelae (Muytjens et al., 1983; Harris and Oriel, 1989; Lucas and Cole, 1990; Van Acker et al., 2001; Dumen, 2010). Moreover, *C. sakazakii* is capable of forming biofilms, commonly found in food processing areas, heightening the risk of infection among vulnerable groups (Iversen et al., 2004; Lehner et al., 2005; Hurrell et al., 2009).

Unfortunately, antibiotic therapies for controlling *C. sakazakii* are limited due to the emergence of resistant strains to many conventional antibiotics (Pitout et al., 1997; Dennison and Morris, 2002; Kilonzo-Nthenge et al., 2012). These strains exhibit resistance to a range of commonly used antibiotics, including ampicillin, cefotaxime, cephalothin, and ceftriaxone. Such resistance not only complicates treatment options but also elevates the risk of potentially severe public health outbreaks (Kim et al., 2007; Zuber et al., 2008). Given these challenges, there are urgent needs to explore new approaches for controlling *C. sakazakii*.

Bacteriophage therapy presents a promising alternative to antibiotics. Specially, phages with lytic life cycle are of significant interest because they target and kill specific bacterial strains without combining their DNA with the host genetic materials (Hagens and Loessner, 2007; Strauch et al., 2007). Several studies have demonstrated the efficacy of phage therapy against *C. sakazakii* infection. For example, the application of *C. sakazakii*-infecting phage CR5 to artificially contaminated infant formula resulted in complete inhibition of bacterial growth at multiplicity of infection (MOI) of 10^5^ (Lee et al., 2016). Additionally, Wang et al. reported that the new *C. sakazakii* phage JK004 significantly reduced bacterial counts in an infant formula milk at 37°C (Wang et al., 2022). However, such phage treatments that involve only a single type of phage may often lead to the development of phage resistance. This issue can be addressed by introducing a cocktail of multiple phages instead of a single phage. Multiple studies have indicated that phage therapy using phage cocktails can be more effective than individual phages alone, especially in cases where the cocktail includes phages that target distinct receptors on the bacterial surface (Bai et al., 2019; Yang et al., 2020). A phage cocktail has been shown to improve bacterial eradication and delay or prevent the development of phage resistance (Tanji et al., 2004; Tanji et al., 2005; Regeimbal et al., 2016). However, only a few studies have specifically examined phage cocktails targeting *C. sakazakii*.

In this study, we isolated two novel *C. sakazakii*-specific phages, CR8 and S13, from environmental samples. We analyzed their biological and genomic characteristics and developed a phage cocktail containing the two phages. The efficacy of this phage cocktail in controlling *C. sakazakii* was evaluated. Additionally, we characterized a phage-resistant *C. sakazakii* isolate, which exhibited the loss of motility as well as altered LPS structures. The phage-resistant isolate demonstrated reduced fitness in invasion assay, suggesting a trade-off between the development of resistance and bacterial fitness.

## 2 Materials and methods

### 2.1 Bacterial strains and growth condition

Bacterial strains used in this study are listed in Table 1. *Cronobacter sakazakii* ATCC 29544 (FARMER et al., 1980) was used as a host strain for bacteriophage isolation and propagation. All the bacterial strains were cultured in tryptic soy broth (TSB, BD Bacto, Franklin Lakes, NJ) or tryptic soy agar (TSA, BD Difco, Franklin Lakes, NJ) at 37°C for 12 h with an agitation speed of 220 rpm.

**Table 1:** Host range of *C. sakazakii* bacteriophages.

Bacterial strains	Plaque formation^a^ of CR8	Plaque formation^a^ of S13	Source^b^
*Cronobacter sakazakii* ATCC 29544	C	C	ATCC
*Cronobacter sakazakii* ATCC BAA-894	-	C	ATCC
*Cronobacter sakazakii* ES15	-	C	Lee et al. (2012)
*Cronobacter muytjensii* ATCC 51329	C	C	ATCC
*Salmonella Typhimurium* ATCC 19586	-	-	ATCC
*Escherichia coli* K-12 MG1655	-	-	Hayashi et al. (2006)
*Listeria monocytogenes* ATCC 19114	-	-	ATCC
*Bacillus cereus* ATCC 14579	-	-	ATCC
*Cronobacter sakazakii* ATCC 29544 Δ*flgK*	-	C	This study
*Cronobacter sakazakii* ATCC 29544 Δ*flgK* pBAD18::*flgK*	C	C	This study
*Cronobacter sakazakii* ATCC 29544 Δ*rfaC*	C	-	This study
*Cronobacter sakazakii* ATCC 29544 Δ*rfaC* pBAD18::*rfaC*	C	C	This study

a C, formation of clear plaque; −, not susceptible to phage.

b ATCC, American Type Culture Collection.

### 2.2 Isolation and propagation of *Cronobacter sakazakii*-specific bacteriophages

Feces and soil samples were collected from University animal farm of Seoul National University (Suwon, South Korea) for isolation of *C. sakazakii*-targeting bacteriophages. The 25 g samples were homogenized in 225 mL sterile Butterfield’s phosphate-buffered dilution water (0.25 M KH_2_PO_4_ adjusted to pH 7.2 with NaOH) using a blender (BacMixer 400, Interscience Laboratory Inc., France). The 25 mL of each homogenized sample was mixed with 25 mL of 2X TSB and incubated at 37°C for 8 h with 220 rpm agitation. After incubation, the culture was centrifuged (9,000 × *g*, 10 min, 4°C) and the supernatant was filtered using 0.22-μm-pore-size filters (Millipore, Billerica, MA). The 10 mL filtrate was mixed with 50 mL of TSB containing 5 mM CaCl_2_ and 500 μL overnight culture of *C. sakazakii* ATCC 29544 and incubated at 37°C for 8 h with 220 rpm agitation, followed by centrifugation and filtration as described above. This supernatant was used for plaque assay. Tenfold serial dilutions of the last filtrate were dotted on the bacterial lawn (5 mL of 0.4% soft TSA containing 100 μL of overnight culture of *C. sakazakii* ATCC 29544 and being poured into TSA plate) and the plate was incubated overnight at 37°C to confirm the presence of bacteriophages via formation of plaques. Each single plaque on dotted or overlaid plate was picked with a sterile tip and dissolved in sterilized sodium chloride-magnesium sulfate (SM) buffer (50 mM Tris–HCl, pH 7.5, 100 mM NaCl, 10 mM MgSO_4_·7H_2_O). This process was repeated five times to isolate a single bacteriophage.

To propagate and concentrate the isolated phages, a culture of *C. sakazakii* ATCC 29544 with an optical density at 600 nm = 0.5 was incubated with SM buffer containing a single phage plaque and the incubation was carried out at 37°C until the culture became clear. After centrifugation of the cleared culture (15,000 × *g*, 10 min, 4°C), the supernatant was filtered with 0.22-μm-pore-size filter to remove cell debris and subsequently precipitated by the addition of polyethylene glycol (PEG) 6,000 (Junsei chemical, Japan) dissolved in 1 M NaCl solution at 4°C for 12 h. Centrifuged and precipitated phages were dissolved in SM buffer and the phage particles were separated by cesium chloride (CsCl) density gradient ultracentrifugation (25,000 rpm, 2 h, 4°C), followed by dialysis using SM buffer and storage at 4°C.

### 2.3 Thermal and pH stability analysis

To determine the stability of CR8 and S13 under various temperature and pH conditions, each phage (~1 × 10^8^ PFU/mL) was incubated overnight at varying temperatures (4–75°C) and pH values (2 to 12). Thermal stability was estimated by incubating 1 mL of phage lysate (~10^8^ PFU/mL) across a temperature range from 4 to 80°C for 1 h. The stability of phages across different pH levels was assessed by exposing the phage lysate to SM buffers with varied pH values for 1 h. The viability of both phages was determined by measuring the concentration of the remaining activated phages using a spotting assay.

### 2.4 Transmission electron microscopy (TEM)

Phage CR8 and S13 stocks were diluted (about 1×10^10^ pfu/ml) with SM buffer and placed on carbon-coated copper grids. Following the removal of the excess phage suspension, negative staining was performed by adding 2% aqueous uranyl acetate (pH 4.0). The grids were examined by TEM (LEO 912 AB; Carl Zeiss, Germany) at 80 kV at the National Academy of Agricultural Science (Suwon, South Korea). Based on the examined morphologies, each phage was classified according to the guidelines of the International Committee on Taxonomy of Viruses (Fauquet et al., 2005).

### 2.5 Genome sequencing and bioinformatics analysis

Bacteriophage genomic DNA was extracted from the phage lysate according to Lambda DNA extraction protocol using the Phase lock gel (PLG; 5 PRIME, Hamburg, Germany). Whole-genome sequencing of the extracted phage DNA was carried out with a Genome Sequencer FLX (GS-FLX) titanium sequencer (Roche, Mannheim, Germany) and assembled with Newbler v2.3 (Roche) at Macrogen Inc., South Korea. Open reading frames (ORFs) were predicted using GeneMarkS (Besemer et al., 2001), Glimmer v3.02 (Delcher et al., 2007), and FgenesB softwares (Softberry, Inc. Mount Kisco, NY, USA), and ribosomal binding sites were identified using RBSfinder (J. Craig Venter Institute, Rockville, MD, USA). BLASTP (Altschul et al., 1990) and InterProScan (Zdobnov and Apweiler, 2001) programs are employed to annotate predicted ORFs, and the sequencing and annotation data were processed by Artemis14 (Carver et al., 2008). The CGView server program was used to generate a circular genome map of the phages (Stothard and Wishart, 2005). Sequence manipulations and genomic analysis were performed using CLC Genomics work-bench version 23 on a workstation at the Biopolymer Research Center for Advanced Material, Sejong University.

Phylogenetic analysis of amino acid sequences of the large subunit of the phage terminase was conducted. Sequence alignment was performed using MUSCLE, and the evolutionary distances among phages were calculated (Edgar, 2004). The phylogenetic tree was constructed using the neighbor-joining method in MEGA 11.0.13, with 1,000 bootstrap replicates (Saitou and Nei, 1987; Tamura et al., 2021).

For genomic comparison between phage CR8 and S13, their genomes were aligned using BLASTn and the results were visualized by Easyfig (Sullivan et al., 2011).

### 2.6 Construction of candidates for phage receptor deletion mutants and complementation

*Cronobacter sakazakii* ATCC 29544 derivatives with deletion of *flgK, rfaC, fhuA, btuB, lamb,* or *ompC* genes were constructed using the one-step gene inactivation method as described previously (Datsenko and Wanner, 2000). Briefly, the kanamycin resistance (Km^r^) cassette from plasmid pKD13 was amplified by PCR using primers specific for each gene. Sequences of all primers for these constructs are listed in Table 2. The resulting PCR products were used to transform into *C. sakazakii* ATCC 29544 strain containing pKD46, and and integrated into the *flgK, rfaC, fhuA, btuB, lamb,* or *ompC* genes. Subsequently, the Km^r^ cassette was removed by introducing plasmid pCP20. For complementation of the deletion mutations, each gene of *C. sakazakii* ATCC 29544 was amplified by PCR using the primers containing restriction site (HindIII and NheI). The PCR products were digested with HindIII and NheI and ligated into HindIII/NheI-digested pBAD18 vector (Guzman et al., 1995). Plasmid constructs were confirmed by sequencing, and transformed into each deletion mutant strains. The protein synthesis was induced by the addition of 0.2% arabinose (final concentration) and phage infectivity was examined by spotting assay.

**Table 2:** Primers used in this study.

Gene	Primer name	Oligonucleotide sequence 5′ – 3′^a^^,^^b^
*flgK*	flgK-red-F	CGC ATG TTC TGC TGA TAC ATC ATT TGT GTA CTA ATA CGC ATC GTT CGG TTC CCT GTG TAG GCT GGA GCT GCT TCG
	flgK-red-R	GCG CTG CCG ATA ATT ATC GTC AGG ACC CGC ATA TGA ATG TTC AAA AGG AAC CTC CAT TCC GGG GAT CCG TCG ACC
	flgK-confirm-F	TAT TTC AGC CAG GTA CTC TGC GAG TCG GTA
	flgK-confirm-R	CTC GAC GCT GTT CTG AAA CCA CTC AAG TTC
	flgK-comple-F-HindIII	GTG TAC TAA T**AA GCT T**CG TTC GGT TCC CTG
	flgK-comple-R-NheI	TAA GC**G CTA GC**G ATA ATT ATC GTC AGG ACC
*rfaC*	rfaC-red-F	AAC GGA TGT TTC CCC GCA AAG CCA GGG ACG CAG TTG TTC AAA AAC GGT AGC GGC GTG TAG GCT GGA GCT GCT TCG
	rfaC-red-R	TGC CCG CGT TCT GGA GAC GCT CAA CGA ACT GCT GCT GAA CGA GGA AGC CTG ACG GAT TCC GGG GAT CCG TCG ACC
	rfaC-confirm-F	AGC AGG GAG AGG TCA ACA ATA CGT
	rfaC-confirm-R	AGG CTA TCA CCA GAG TCT CAT CGA
	rfaC-comple-F-SalI	AAA C**GT CGA C**GT GGT GGC GCA GCT T
	rfaC-comple-R-EcoRI	AAA A**GA ATT C**TG GAG ACG CTC AAC GAA CTG
*fhuA*	fhuA-red-F	TCA AAC AGG TTA TTG ACG TTT AAG GCG ACA GAC GAG CCC GGC AGG CCT AAA CGC GTG TAG GCT GGA GCT GCT TCG
	fhuA-red-R	TAG CAT GGC GCG TTC CAC TCA CAC TCA GAT CAA TAC CAG GAT TTG CAG ACT GGC GAT TCC GGG GAT CCG TCG ACC
	fhuA-confirm-F	GCG TAG TCT TTG TAG CAG CTA GAG
	fhuA-confirm-R	CAA CCT TTC GCA TAT CAT CTC GGG
*btuB*	btuB-red-F	ACC AGA ATG GTT GGG CGG GCC TCA GAA GGT GTA GCT GCC TGA CAA CGT GTA TTC TTG TAG GCT GGA GCT GCT TCG
	btuB-red-R	GCA TCT CGC GCG CTT ATC GCG TTC TAT TAA GTG CGC CTG CGG CAT CCT ATA CGT TAT TCC GGG GAT CCG TCG ACC
	btuB-confirm-F	TCA TAA ACA GAA AGC CCA CCC ACG
	btuB-confirm-R	AAG TCC TCA TCC GTA ACA CAC CTC
*lamB*	lamB-red-F	GAG ATA GAA TGA TGA TAA CTC TGC GTA AAC TCC CTC TGG CTG TGG CCG TGA TGG CTG TAG GCT GGA GCT GCT TCG
	lamB-red-R	TAC CAC CAG ATT TCC ATC TGG GCA CCG AAG GTC CAC TCA TCA TTG TCG CCA CGG CAT TCC GGG GAT CCG TCG ACC
	lamB-confirm-F	CCC CGC TTA CAA AGA AAA GC
	lamB-confirm-R	CTT TCG CCC CTC TTG TTA CA
*ompC*	ompC-red-F	TCG GAC AAT GGA TTT GCC CGC TAG TTC CCT GAA TTA GTG AGC AGT GGC AAT AAT ATG TAG GCT GGA GCT GCT TCG
	ompC-red-R	GGA GCC CGC AGG CTC CTT TTG CAC ATC AGG TCG GGG ATT AGA ACT GGT AAA CCA GAT TCC GGG GAT CCG TCG ACC
	ompC-confirm-F	CTG TTG GAT TAT TCG GCT CC
	ompC-confirm-R	CAC ACG TTT CTC CTC TGT AAC
pBAD18	pBAD18-F	GTC CAC ATT GAT TAT TTG CAC G
	pBAD18-R	CAG GCT GAA AAT CTT CTC TCA T

a Sequences of priming sites in pKD13 were underlined.

b Restriction sites are bold.

### 2.7 Adsorption assay

*Cronobacter sakazakii* ATCC 29544 wild-type or deletion mutant strains were grown exponentially until OD_600_ reached to 1.0 to 1.5 and 900 μL of the culture was aliquoted into 6 microtubes (900 μL culture per 1.75-ml tube). Each culture was infected by phage (MOI = 0.01) with 5 min intervals and incubated until the first infected tube was incubated for 20 min. After incubation, all tubes were centrifuged (16,000 rpm, 1 min, 4°C) and the supernatant was filtrated (0.22-μm-pore-size filter) to remove bacterial cells. Overlay assay was carried out as previously described (Kim and Ryu, 2011), to determine the number of unadsorbed phage particles. For control, phage was added to fresh TSB without host bacteria.

### 2.8 Host range analysis

An overnight culture of each tested bacteria (100 μL) was inoculated into 5 mL of 0.4% soft TSA and mixed by gentle vortexing. The mixture was poured into the 1.5% TSA plate (with 50 μg/mL ampicillin or kanamycin, if necessary) and placed at room temperature (RT) for 20 min to allow the agar to solidify. Then, 10 μL of tenfold serial dilutions of phage stock was dotted on the bacterial lawn. The plates were dried at RT for 20 min, followed by overnight incubation at 37°C. The susceptibility of the tested bacteria to phages was examined on the following day.

### 2.9 Growth inhibition assay

An overnight culture of *C. sakazakii* ATCC 29544 was 1% subinoculated into 50 mL of fresh TSB medium and incubated at 37°C with agitation for 1.5 h (OD at 600 nm = 0.4 ~ 0.6). Phages were added to the culture at an MOI of 1. Bacterial growth was monitored by measuring the OD_600_ every hour after phage infection. An uninfected culture was used as a negative control.

A phage cocktail consisting of phage CR8 and S13 was evaluate for their ability to inhibit the growth of host bacteria. The phages CR8 and S13 were added to the bacterial culture prepared as above at an MOI of 1 each. To count the number of viable cells, samples taken from the culture were centrifuged (15,000 rpm, 1 min) to remove free phage and the cell pellet was resuspended in same volume of phosphate-buffered saline (PBS, GeneDEPOT, Barker, TX, USA) (pH 7.4). Then, it was 10-fold diluted with PBS, plated onto TSA plate, and followed incubation at 37°C for 12 h.

### 2.10 Isolation and characterization of phage cocktail-resistant mutants

To isolate phage cocktail-resistant mutants, the high-titer phage cocktail overlay assay was conducted as described previously (Capparelli et al., 2010). Briefly, 100 μL of each phage (about 10^10^ PFU/mL, for each) and the same volume of overnight bacterial culture were added to molten 5-ml soft TSA (0.4% agar). The mixture was poured onto TSA plate and incubated at 37°C until resistant colonies appeared. Three colonies were picked and streak-purified 20 times on fresh TSA plate in the absence of phages. One of them was selected for further study and the maintenance of phage resistance was certified by spotting assay.

### 2.11 Motility assay

One microliter of the overnight culture was injected onto soft-agar motility plates (TSA medium containing 0.3% agar) to allow bacteria to swim in the plates. The plates were dried for 30 min at room temperature and incubated for 8 h at 37°C. As a positive and negative control, *C. sakazakii* ATCC 29544 wild type strain and flagella (Δ*flgK*) defective mutant strain were used, respectively.

### 2.12 LPS extraction

LPS was extracted from bacteria grown overnight using hot phenol-water extraction as previously described (Kim and Ryu, 2012; Kim et al., 2018). The extracted LPS was subjected to deoxycholate polyacrylamide gel electrophoresis (DOC-PAGE) followed by fluorescent staining using the Pro-Q® Emerald 300 Lipopolysaccharide Gel Stain Kit (Molecular Probes, Cat. No. P20495; Eugene, Oregon, USA) according to manufacturer’s instructions. The gel was visualized under 300 nm UV by the Gel doc™ EZ System (Bio-rad). As a positive and negative control, *C. sakazakii* ATCC 29544 wild type strain and LPS (Δ*rfaC*) defective mutant strain were used, respectively.

### 2.13 Autoaggregation assay

Autoaggregation assay was conducted as previously described (Kim et al., 2018). Briefly, bacteria were cultured overnight, subcultured in fresh TSB medium at 1%, and then incubated at 37°C with constant shaking. Mid-exponentially grown bacteria were diluted with fresh TSB broth to achieve an OD_600_ of 1.5 in glass tubes and left at room temperature.

### 2.14 Invasion assay

Bacterial invasion ability into mammalian Caco-2 cells was assessed using a modified gentamicin protection assay (Kim et al., 2010). Bacteria were cultured overnight, then transferred to fresh LB medium at a 1% concentration and incubated at 37°C for 3 h with 220 rpm agitation. The OD_600_-adjusted bacterial cells were pelleted, resuspended in 400 μL of EMEM for infection and used to infect monolayered cells. The infection was carried out for 1.5 h in the presence of 5% CO_2_ and washed 3X with PBS. Gentamicin was treated to eliminate any bacteria that were adhering to cells for 1.5 h and washed 3X with PBS. Cells were then lysed with 1% Triton X-100 (500 μL/ well) for 15 min, cell lysates were serially diluted and plated onto LB plates to enumerate CFUs. Relative invasion capability was calculated as described previously (Kim et al., 2010) using following formula:


$$\frac{\text{the}\;\text{No}.\;\text{of}\;\text{survived}\;\text{mutant}\;\text{bacteria}}{\text{the}\;\text{No}.\;\text{of}\;\text{survived}\;WT\;\text{bacteria}}$$

#### 2.14.1 Data processing and statistical analysis

Statistical analysis was performed using Student’s unpaired t-test in Prism (version 10.3.0), with significance represented as ****p < 0.0001 for invasion into host cells.

## 3 Results

### 3.1 Isolation of phages and their host range determination

Out of five *C. sakazakii*-infecting phages isolated from environmental samples, the phages CR8 and S13 were selected for this study due to their superior lytic activity against *C. sakazakii* and their ability to target different host receptors, ensuring a more effective and robust phage cocktail. The two phages formed clear plaques without halos on the lawn of *C. sakazakii* ATCC29544. The stability of CR8 and S13 under various temperature and pH conditions were evaluated. Both phages maintained high thermal stability from 4°C to 45°C, but a significant decrease was evident at 55°C, with a notable drop in titer for both CR8 and S13. In a wide pH range (2 to 12), the phages remained highly stable, indicating that both CR8 and S13 phages have robust pH tolerance, which may be advantageous for various application where pH fluctuations occur (Supplementary Figure S1). Their host range studies determined by spotting assay on strains of *Cronobacter*, *Escherichia coli, Salmonella Typhimurium*, *Listeria monocytogenes*, and *Bacillus cereus* showed that these phages formed plaques only on *Cronobacter* genus (Table 1).

### 3.2 Morphological analysis of phage CR8 and S13 by TEM

**Figure 1:**
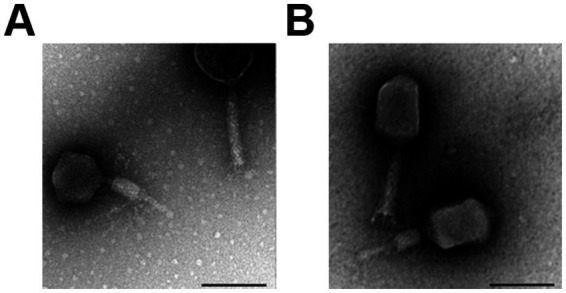
Morphology of phage CR8 and S13. TEM images of phage CR8 **(A)**, S13 **(B)** with contractile tail. Scale bar, 100 nm.

Morphological analysis revealed that both phages have a characteristic of the family *Caudoviricetes* with a contractile tail (Figure 1). Phage CR8 has an isometric head with the mean diameter of 78.6 ± 1.7 nm, and phage S13 has longish head with the major axis of 103.7 ± 4.1 nm and the minor axis of 74.7 ± 6.0 nm. Their tail length is similar to each other – non-contracted tail and contracted tail length of phage CR8 are 122.1 ± 2.3 nm and 58.1 nm, respectively, and those of phage S13 are 112.0 ± 2.1 nm and 55.0 ± 2.1 nm (Figure 1). According to the International Committee on Taxonomy of Viruses (ICTV, https://ictv.global/, accessed on 20 August 2024), CR8 is predicted to belong to the *Certrevirus* genus and S13 shares genetic homologies with phages in the *Straboviridae* family, genus *Slopekvirus*.

### 3.3 Bacteriophage genome analysis

**Figure 2:**
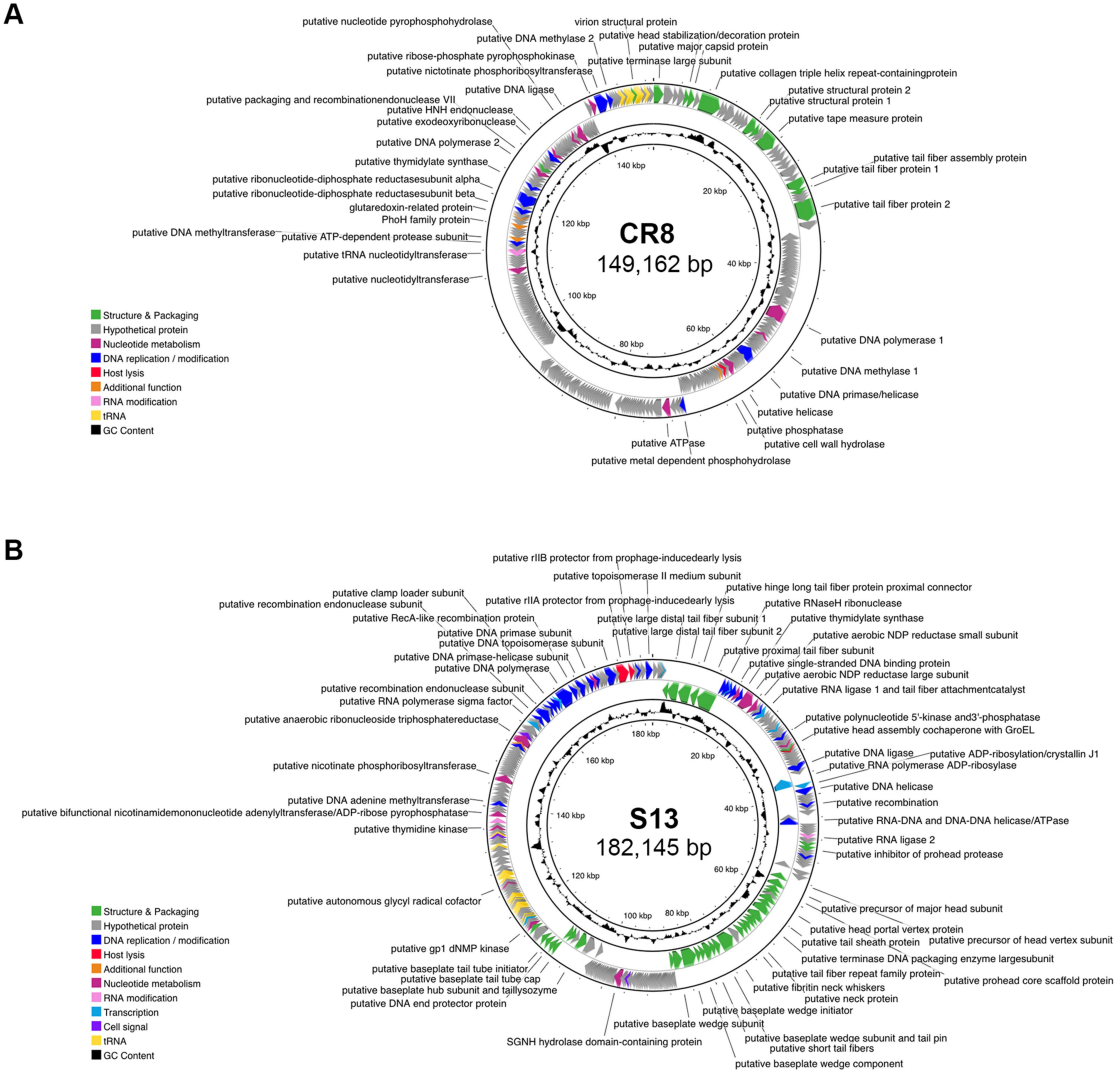
Genomic features of phage CR8 and S13. Genome maps of CR8 **(A)** and S13 **(B)**. Predicted ORFs in the genome were annotated and colored by functional groups as follows: phage structure and packaging (green), nucleotide metabolism (magenta), DNA replication/modification (blue), host lysis (red), RNA modification (pink), transcription (teal), cell signal (purple) and additional function (orange).

Whole genome analysis revealed that the genome sizes of CR8 and S13 are 149,162-bp and 182,145-bp, respectively and the GC contents are 50.8 and 40.2%, respectively. Phages CR8 and S13 contain 269 ORFs with 17 tRNAs and 270 ORFs with 25 tRNAs, respectively (Table 3; Figure 2). No genes related to virulence and antibiotic resistance were identified in the genome of both phages, demonstrating their safety in application. The majority of predicted genes encoded hypothetical proteins (86.6% for CR8 and 64.1% for S13). Additional details on each ORF are provided in the Supplementary Table S1. Meanwhile, phage CR8 genome shares 97% nucleotide identity with that of phage CR3, a *C. sakazakii* infecting phage (Shin et al., 2012), but they did not have similarity with other phages genomes publicized in GenBank database. Phage S13 genome showed little sequence homology with well-known T4 phage. Additionally, no sequence similarity between CR8 and S13 was observed at the nucleotide level (Supplementary Figure S2).

**Table 3:** Genomic characteristics of phage CR8 and phage S13.

Characteristics	Bacteriophages	
	CR8	S13
Length (bp)	149,162	182,145
Overall G + C content (%)	50.8	40.2
No. of annotated genes	269	270
Percentage of hypothetical proteins (%)	86.6	64.1
Avg gene length (bp)	497	632
Gene coding content (%)	89.6	93.7
No. of tRNAs	17	25

The ORFs were categorized into eight functional groups; structure/packaging, DNA replication/modification, host lysis, nucleotide metabolism, RNA modification, transcription, cell signal and other groups. Genes related to lysogenic functions such as integrase, transposase, excisionase, repressor, and the genome attachment site (attP) were absent in the genome of CR8 and S13, suggesting that the two phages are likely lytic phages. Phage CR8 has two genes encoding tail fiber proteins, CR8_028 and CR8_033, which are likely receptor binding protein (RBP) targeting the flagella of *C. sakazakii*. Meanwhile, phage S13 possesses genes encoding for multiple tail fiber subunits and putative RBP (S13_008), which likely targets the LPS of *C. sakazakii*. Interestingly, S13 contains a gene encoding putative RNA polymerase sigma factor which is utilized in gene expression at the middle and late stages (Reznikoff et al., 1985), suggesting their potential role in preferential transcription of phages genes over host genes. Genes related to host lysis, such as holin and endolysin, were not detected in the phage CR8 genome, but it contains a cell wall hydrolase (CR8_090), which shows 99% amino acid sequence identity with an endolysin of phage CR3. A gene encoding a putative lysozyme (S13_190) was identified in the phage S13 genome, but its amino acid sequence showed no homology with any known lysozyme from *Cronobacter* phages. It exhibited 71 and 69% homology with the lysozyme from *Citrobacter* phage Ci1 and *Shigella* phage Esh27, respectively.

The phylogenetic analysis of the two phages was conducted based on their large subunit of terminase (Supplementary Figure S3). The resulting trees include *Cronobacter* phages, alongside other phages from different bacterial hosts. CR8 closely aligns with *Pectobacterium* phage vB_PcaM_P7_Pc, a member of the genus *Cetrevirus*. Phage vB_PcaM_P7_Pc exhibited myovirus-like morphology with lytic life cycle. Phage S13 is positioned near the *Cronobacter* phage LPCS28, which is also lytic phage. The findings highlight potential of phages CR8 and S13 for use as biocontrol or as therapeutic agents.

### 3.4 Identification of phage receptor

**Figure 3:**
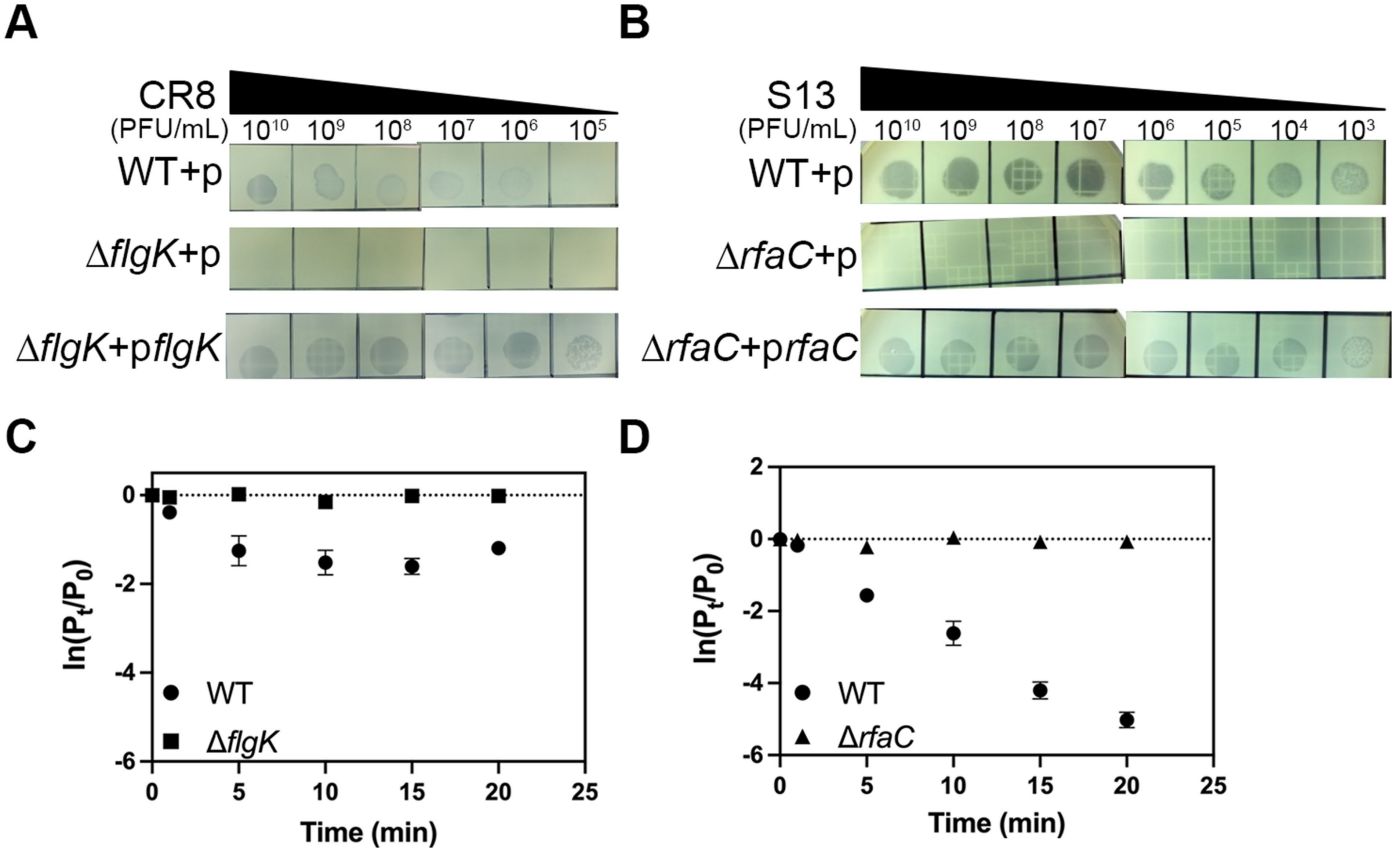
Flagella and LPS are receptors for CR3 and S13, respectively. The sensitivity of the bacteria to CR3 **(A)** and S13 **(B)** phages was determined by spotting assay. Complementation of flagella and LPS completely restored the phage susceptibility. Noted strains are WT + p, wild type harboring pBAD18; Δ*flgK* + p, Δ*flgK* harboring pBAD18; Δ*flgK* + p*flgK*, Δ*flgK* expressing *flgK*, Δ*rfaC* + p, Δ*flgK* harboring pBAD18; Δ*rfaC* + p*rfaC* Δ*rfaC* expressing *rfaC*. **(C,D)** adsorption kinetics of phage CR8 **(C)** and S13 **(D)** against *C. sakazakii* ATCC 29544 (circle), Δ*flgK* (square) and Δ*rfaC* (triangle). All bacterial strains grown exponentially were challenged with each phage at an MOI of 0.01. Data displayed are mean ± SEM for three biological replicates. P_t_, phage titer (PFU/ mL) at the indicated time; P_0_, initial phage titer.

To identify bacterial components that are required for phage infection, we decided to test six receptors known in Gram-negative bacteria (Rakhuba et al., 2010). To this end, we constructed deletion mutants of *C. sakazakii* ATCC 29544, each lacking one of the followings: flagella (Δ*flgK*), LPS (Δ*rfaC*), outer membrane transporter for ferrichrome (Δ*fhuA*), vitamin B12 (Δ*btuB*), maltose (Δ*lamB*), and outer membrane porin OmpC (Δ*ompC*). These mutants were challenged to test susceptibility to specific phages. Phage CR8 was unable to form any plaques on the lawns of Δ*flgK* deletion mutant and phage S13 failed to form plaques on the lawns of Δ*rfaC* deletion mutant (Figures 3A,B). However, the infectivity of both phages to *C. sakazakii* ATCC 29544 was fully restored when *flgK* and *rfaC* genes were reintroduced into their respective mutants (Figures 3A,B). To determine whether these genes are required for the initial step of phage infection, phage adsorption assay was conducted with the WT and deletion mutants. The ability of phage CR8 to bind to its host receptor decreased in the absence of *flgK* (Figure 3C). Most of the phage S13 efficiently bound to the WT strain within 20 min, while its binding to the Δ*rfaC* deletion mutant was completely abolished (Figure 3D). These results indicate that flagella and LPS serve as receptors for the phage CR8 and S13, respectively.

### 3.5 Bacterial challenge assay of a phage cocktail targeting two different host receptors

**Figure 4:**
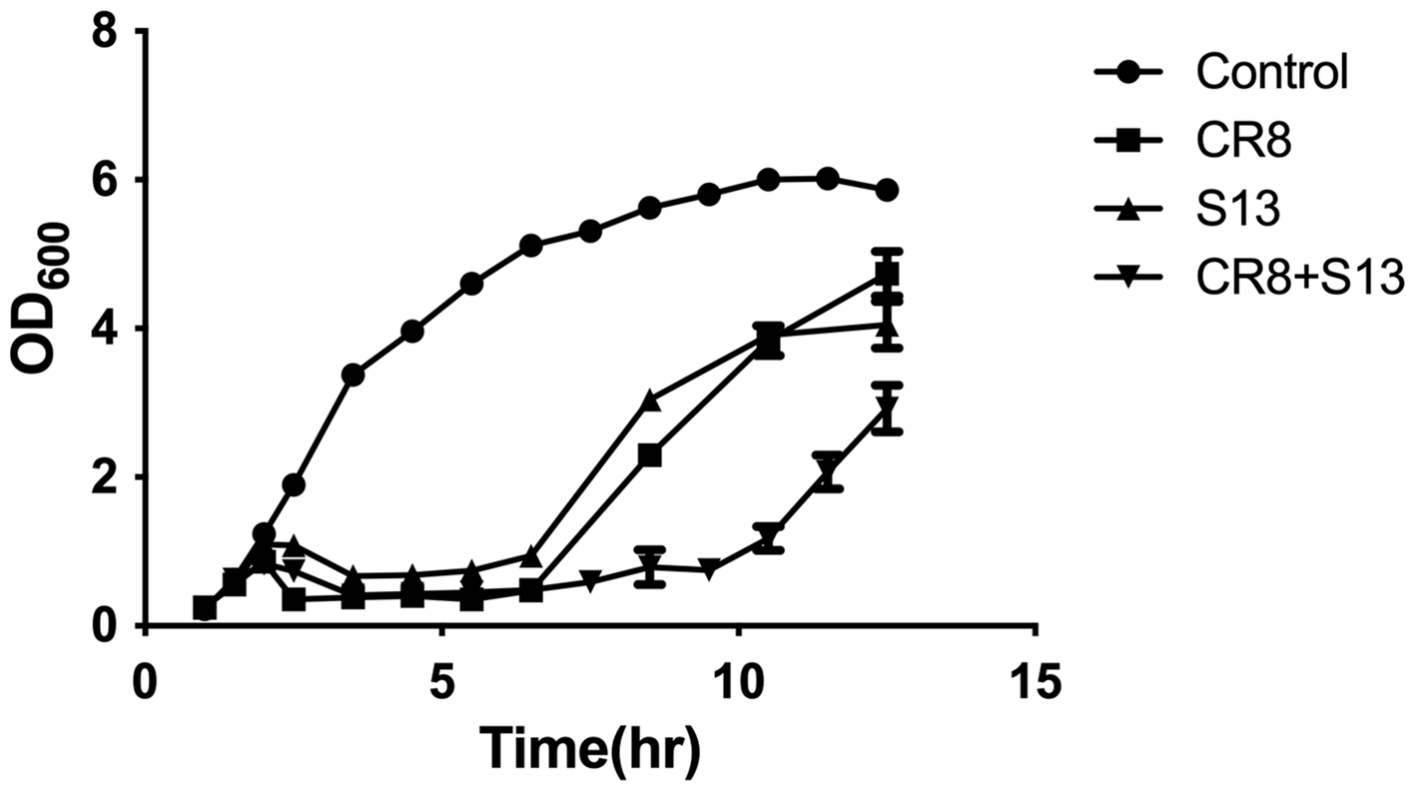
Bacterial challenge assay with phages CR8 and S13 against *C. sakazakii* ATCC 29544. Each phage or a phage cocktail was added at a MOI of 1 to the bacterial culture after 1.5 h incubation. Bacterial growth was determined by measuring absorbance at 600 nm hourly. Data shown and error bars are means ± SEM for three biological replicates at 1 h increments.

The ability of a phage cocktail to inhibit the growth of host bacteria was evaluated using bacterial challenge assays. Phage CR8 and phage S13 showed similar ability to lyse host bacteria with rapid early cell lysis, and the phage-resistance appeared 5 h after infection. On the other hand, a phage cocktail consisting of phage CR8 and phage S13 showed the prolonged period of growth inhibition up to 11 h, resulting in the delayed emergence of the resistant mutant (Figure 4A) and there was a significant reduction in the number of viable cells compared to single-phage treatments (Figure 4B).

### 3.6 Isolation and characterization of phage cocktail-resistant mutants

**Figure 5:**
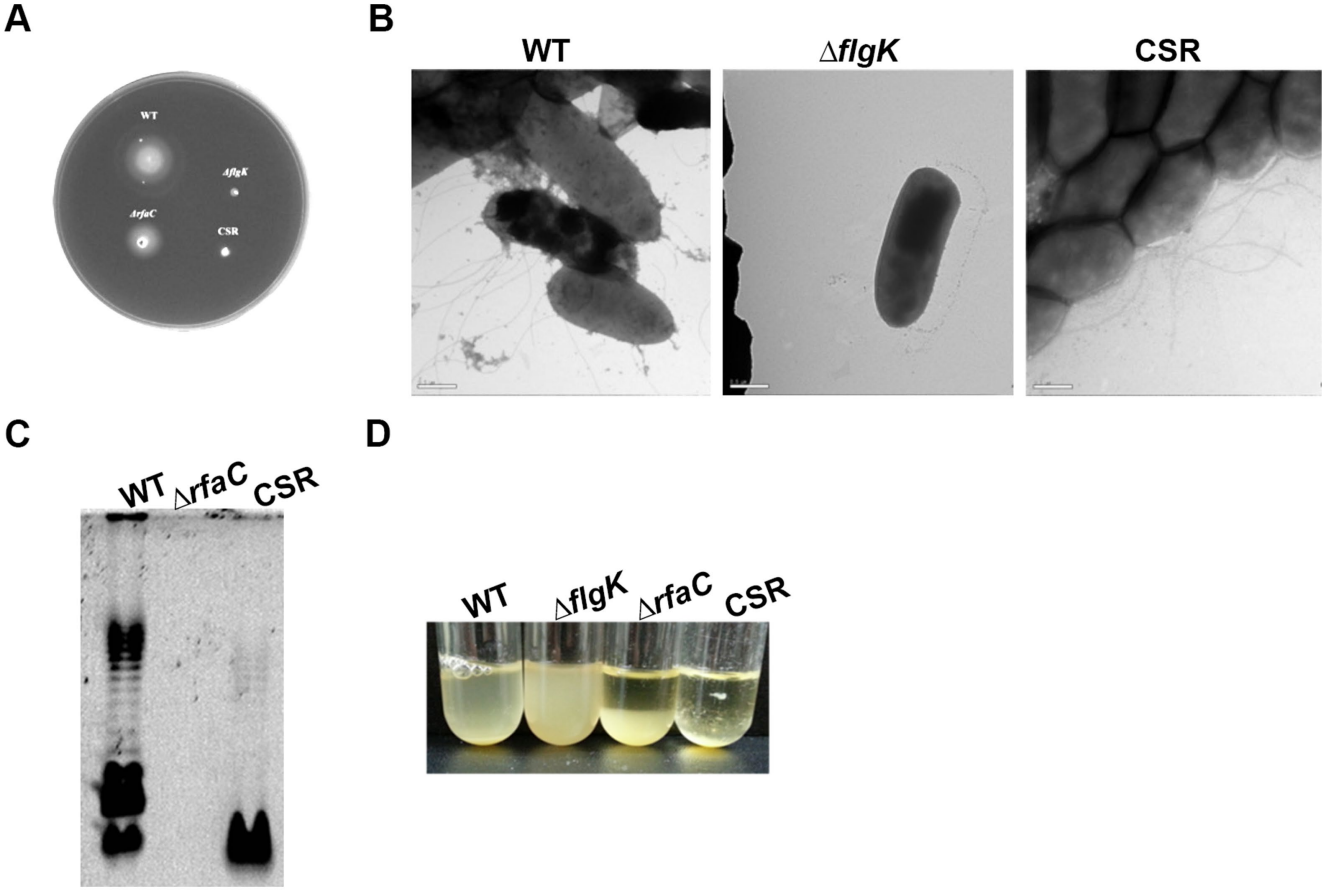
Characterization of CSR strain resistant to a phage cocktail infection. **(A)** Motility of *C. sakazakii* strains on semisolid agar plates: WT, Δ*flgK*, Δ*rfaC* and CSR, respectively. **(B)** TEM analysis. Scale bars, 500 nm. **(C)** LPS profiles. LPS fractions were extracted using hot phenol-water method, fractionated by DOC-PAGE and fluorescently stained. **(D)** Autoaggregation of the CSR strain. Bacterial cultures of WT, Δ*flgK*, Δ*rfaC*, and CSR strain were diluted at an OD_600_ of 1.5 using fresh TSB broth and incubated statically at room temperature. The data displayed are a representative of three biological replicates.

Three phage cocktail-resistant *C. sakazakii* colonies were picked from high-titer overlay plates and streak-purified 20 times onto fresh TSA plates in the absence of phages to isolate a single mutant strain. At each streaking-purifying step, the newly formed colonies were subjected to spotting assay to confirm phage resistance and a single colony that exhibited consistent resistance across generations was selected for further study and named CSR strain. Given that CR8 and S13 target flagella and LPS as receptors, we hypothesized that the resistant mutant might lack flagella and LPS or exhibit loss of motility and altered LPS structure. To test these hypotheses, motility and flagella structure were examined. The CSR strain was defective in motility like Δ*flgK* deletion mutant, yet still displayed flagella on its surface (Figures 5A,B). LPS structure of CSR strain differed from that of WT and Δ*rfaC* deletion mutant; some parts of inner core and O-antigen regions of LPS were absent in CSR strain (Figure 5C). It should be noted that nothing was extracted in Δ*rfaC* deletion mutant, which produces a lipophilic moiety (lipid A-Kdo2; rough-type of LPS) (Figure 5C; Sirisena et al., 1992), because the hot phenol-water method is selective for smooth types of LPS over rough types (Henderson et al., 2013). Previous studies have demonstrated that the alteration in LPS leads to autoaggregation in *C. sakazakii* (Wang et al., 2012; Kim et al., 2018). Consistent with these findings, CSR cells precipitated more quickly than WT cells when left in broth culture at room temperature, an indicative of enhanced autoaggregation (Figure 5D).

### 3.7 Decreased fitness in virulence resulting from acquiring phage-resistance

**Figure 6:**
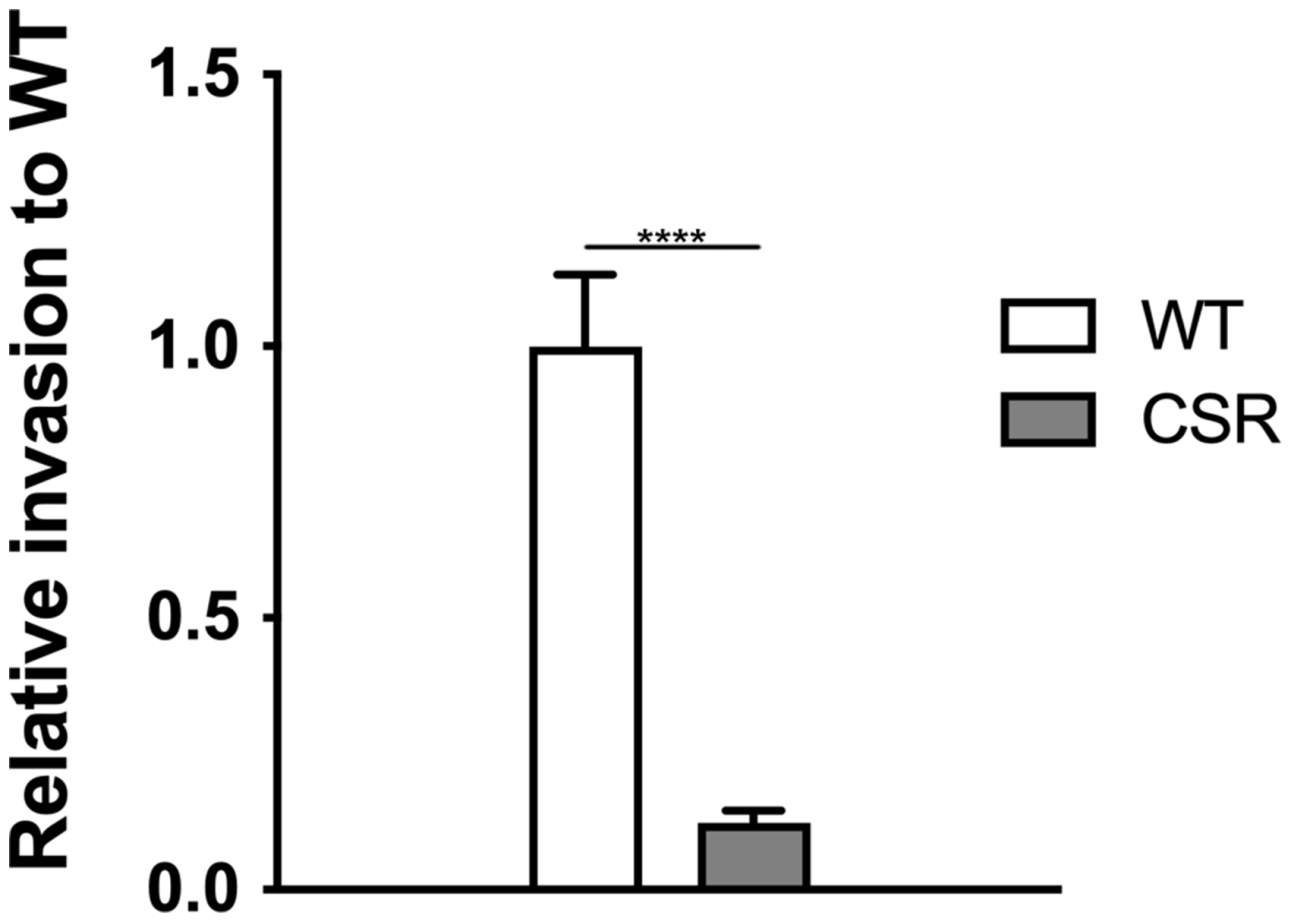
Attenuation in invasion resulted from the acquisition of phage resistance. Invasive ability into human epithelial cells, Caco-2. Confluent monolayers of Caco-2 epithelial cells were challenged with WT *C. sakazakii* or the CSR strain, incubated for 1.5 h, followed by gentamicin treatment at a concentration of 100 μg/mL for a further 1.5 h. The Caco-2 cells were lysed with 1% TritonX-100 to harvest the intracellular bacteria. The data shown and error bars are mean ± SEM for three biological replicates. Statistical significance was conducted using unpaired two- tailed Student’s t test and significance displayed as ****p < 0.0001.

Considering that the development of phage resistance often incurs fitness costs, including reduced virulence and heightened vulnerability to antimicrobials (Chart et al., 1989; Chan et al., 2016; Gordillo Altamirano et al., 2021; Salazar et al., 2021; Wang et al., 2021) or immune clearance (Roach et al., 2017), we aimed to evaluate invasion capability of the CSR strain to mammalian intestinal epithelial Caco-2cells. Consistent with previous studies, invasiveness of the CSR strain was significantly reduced by 90% compared to WT cells (Figure 6).

## 4 Discussion

*Cronobacter sakazakii* has been detected in various foods and environmental sources (Kandhai et al., 2004), but it is difficult to destroy the pathogen without affecting the quality of foods (Dumen, 2010). When the foods were stored improperly, the pathogen can propagate to cause the disease in immunocompromised human. Outbreaks of disease associated with *C. sakazakii* have occurred sporadically and are particularly dangerous to infants, suggesting that new measures to protect infants from the pathogen are needed. In the food safety aspect, bacteriophages have been studied and evaluated with promising results due to their host specificity (Garcia et al., 2008). The specificity of phage is related to their adsorption on bacterial cell surface, especially receptors (Rakhuba et al., 2010). In the present study, we isolated novel phages which utilize different receptor each other and characterized them to construct effective phage cocktail.

Phage CR8 and phage S13 belong to the *Caudoviricetes* family, and interestingly, the head of phage S13 is not isometric; rather, it is slightly elongated (Figure 1). This prolate head is unusual because isometric heads are in a majority (85%) of the tailed phages (Calendar and Abedon, 2005). In our genome analysis, phage CR8 has a genome size of 149,162 bp, and phage S13 has 182,145 bp. The larger head size of S13 compared to CR8 corroborates previous findings that phage head size varies proportionally with genome size (Pariza and Iandolo, 1974). Interestingly, the majority of genes in the phage CR8 and S13 encode hypothetical proteins, suggesting the phages are quite distinct from those previously reported. Additionally, limited genetic information is available on *C. sakazakii* phages, indicating the need for further research in this area.

Phage cocktail containing phages that target different host receptors has been effective in phage therapy. Bai et al. reported that a phage cocktail containing three *Salmonella* phages, each targeting different host receptors such as flagella, O-antigen and BtuB, significantly reduced the development of bacterial resistance (Bai et al., 2019) Similarly, a phage cocktail comprising five *Pseudomonas* infecting phages, each targeting distinct receptors, was shown to effectively constrain the emergence of phage resistant *Pseudomonas* (Yang et al., 2020). Treatment of the phage cocktail containing phage CR8 and S13 delayed the development of resistant bacteria by several hours compared to the treatment with each single phage, demonstrating that a phage cocktail could be a powerful strategy to control *C. sakazakii*. Bacteria can evolve to gain resistance to phage through a range of mechanisms (Labrie et al., 2010), notable one of which is loss or modification of receptor, which prevents phage adsorption (Filippov et al., 2011). We observed the loss of motility and the auto-aggregation in CSR, leading us to initially assume that the resistant strains have no flagella and LPS. However, TEM analysis demonstrated that the CSR still possess flagella similar to wild-type strain. These findings suggest that the resistance may arise from events occurring during the phage replication process following adsorption, rather than from the absence of structural receptors such as flagella. It should be noted that the direction of flagellar rotation, in addition to retaining the flagellar structure itself, is required for the *Salmonella* phage iEPS5 to adsorb and successfully infect the host bacteria (Choi et al., 2013). DOC-PAGE analysis of LPS revealed that CSR has defective O-antigen and core region in their LPS (Figure 5C). Incomplete deletion of O-antigen suggest that LPS core domain of *C. sakazakii* contains branched oligosaccharide and that CSR was affected in some branch as reported in *E. coli* K1 strain (Jiménez et al., 2012). It remains unclear which gene in CSR was affected, thus genetic analyses such as whole genome sequencing are necessary for future study.

In the context of Red Queen dynamics, the emergence of bacterial resistance to phages is inevitable. As anticipated, we observed the emergence of mutants resistant to a phage cocktail. Among these, we isolated and characterized a mutant, designated CSR, which exhibited stable resistance to phage infections (Figures 5, 6). Notably, CSR exhibited a loss of motility despite retaining flagellar structure (Figure 5) and displayed alteration in its LPS structures compared to WT strain (Figure 5). It is noteworthy that the emergence of phage-resistant bacteria is considered clinically significant, as they often become susceptible to antibiotics or hypovirulent (Niu et al., 2020; Gordillo Altamirano et al., 2021; Majkowska-Skrobek et al., 2021; Song et al., 2021). Consistent with the fact that phage selective pressure on hypervirulent bacteria reduces virulence by altering bacterial surface appendages, including LPS and capsule (Tang et al., 2023), the CSR isolate showed highly impaired invasion ability into intestinal epithelial cells. This finding supports the potential use of the phage cocktail in this study as a novel antibacterial agent.

## 5 Conclusion

We evaluated the efficacy of a phage cocktail consisting of two distinct phages, CR3 and S13, which target different host receptors, for potential use as a biocontrol agent. Morphological and genomic analyses confirmed that both phages belong to members of the *Caudoviricetes* class with contractile tails. Phage CR8 targets flagella, while phage S13 targets LPS as their respective host receptors. The combination of these two phages significantly delayed the emergence of the resistant mutant by prolonging period of growth inhibition and demonstrated stronger lytic activity compared to individual phage treatments. Notably, we isolated a phage-resistant strain with phenotypic defects in motility and altered LPS structure. The resistant strain exhibited severe impaired invasion ability to human epithelial cells, likely due to a fitness tradeoff between acquisition of the resistance and virulence. Overall, the phage cocktail presents a promising and sustainable alternative to antibiotics for controlling *Cronobacter.*

## Data Availability

The original contributions presented in the study are publicly available. This data can be found at: https://www.ncbi.nlm.nih.gov/genbank, accession numbers KC954774 and KC954775.

## References

[ref1] Altschul S. F. Gish W. Miller W. Myers E. W. Lipman D. J. (1990). Basic local alignment search tool. J. Mol. Biol. 215, 403–410. doi: 10.1016/S0022-2836(05)80360-2 2231712

[ref2] Bai J. Jeon B. Ryu S. (2019). Effective inhibition of *Salmonella Typhimurium* in fresh produce by a phage cocktail targeting multiple host receptors. Food Microbiol. 77, 52–60. doi: 10.1016/j.fm.2018.08.011 30297056

[ref3] Besemer J. Lomsadze A. Borodovsky M. (2001). GeneMarkS: a self-training method for prediction of gene starts in microbial genomes. Implications for finding sequence motifs in regulatory regions. Nucleic Acids Res. 29, 2607–2618. doi: 10.1093/nar/29.12.2607, PMID: 11410670 PMC55746

[ref4] Calendar R. Abedon S. T. (2005). The bacteriophages. Oxford: Oxford University Press.

[ref5] Capparelli R. Nocerino N. Lanzetta R. Silipo A. Amoresano A. Giangrande C.. (2010). Bacteriophage-resistant *Staphylococcus aureus* mutant confers broad immunity against *staphylococcal* infection in mice. PLoS One 5:e11720. doi: 10.1371/journal.pone.0011720, PMID: 20661301 PMC2908692

[ref6] Carver T. Berriman M. Tivey A. Patel C. Böhme U. Barrell B. G.. (2008). Artemis and ACT: viewing, annotating and comparing sequences stored in a relational database. Bioinformatics 24, 2672–2676. doi: 10.1093/bioinformatics/btn529, PMID: 18845581 PMC2606163

[ref7] Chan B. K. Sistrom M. Wertz J. E. Kortright K. E. Narayan D. Turner P. E. (2016). Phage selection restores antibiotic sensitivity in MDR *Pseudomonas aeruginosa*. Sci. Rep. 6:26717. doi: 10.1038/srep26717 27225966 PMC4880932

[ref8] Chart H. Row B. Threlfall E. J. Ward L. R. (1989). Conversion of *Salmonella enteritidis* phage type 4 to phage type 7 involves loss of lipopolysaccharide with concomitant loss of virulence. FEMS Microbiol. Lett. 60, 37–40. doi: 10.1016/0378-1097(89)90073-6, PMID: 2676707

[ref9] Choi Y. Shin H. Lee J. H. Ryu S. (2013). Identification and characterization of a novel flagellum-dependent *Salmonella*-infecting bacteriophage, iEPS5. Appl. Environ. Microbiol. 79, 4829–4837. doi: 10.1128/AEM.00706-13, PMID: 23747700 PMC3754727

[ref10] Datsenko K. A. Wanner B. L. (2000). One-step inactivation of chromosomal genes in *Escherichia coli* K-12 using PCR products. Proc. Natl. Acad. Sci. USA 97, 6640–6645. doi: 10.1073/pnas.120163297, PMID: 10829079 PMC18686

[ref11] Delcher A. L. Bratke K. A. Powers E. C. Salzberg S. L. (2007). Identifying bacterial genes and endosymbiont DNA with glimmer. Bioinformatics 23, 673–679. doi: 10.1093/bioinformatics/btm009, PMID: 17237039 PMC2387122

[ref12] Dennison S. K. Morris J. (2002). Multiresistant *Enterobacter sakazakii* wound infection in an adult. Inf. Med. 19, 533–535.

[ref13] Dumen E. (2010). *Coronabacter sakazakii* (*Enterobacter sakazakii*) only an infant problem. Kafkas Univ Vet Fak Derg 16, S171–S178. doi: 10.9775/kvfd.2010.1949

[ref14] Edgar R. C. (2004). MUSCLE: multiple sequence alignment with high accuracy and high throughput. Nucleic Acids Res. 32, 1792–1797. doi: 10.1093/nar/gkh340, PMID: 15034147 PMC390337

[ref15] Farmer J. J. Asbury M. A. Hickman F. W. Brenner D. J. (1980). *Enterobacter sakazakii*: a new species of “*Enterobacteriaceae*” isolated from clinical specimens. Int. J. Syst. Bacteriol. 30, 569–584. doi: 10.1099/00207713-30-3-569

[ref16] Fauquet C. M. Mayo M. Maniloff J. Desselberger U. Ball L. A. (2005). Virus taxonomy: VIIIth report of the international committee on taxonomy of viruses. Cambridge: Academic Press.

[ref17] Filippov A. A. Sergueev K. V. He Y. Huang X.-Z. Gnade B. T. Mueller A. J.. (2011). Bacteriophage-resistant mutants in *Yersinia pestis*: identification of phage receptors and attenuation for mice. PLoS One 6:e25486. doi: 10.1371/journal.pone.0025486, PMID: 21980477 PMC3182234

[ref18] Garcia P. Martinez B. Obeso J. Rodríguez A. (2008). Bacteriophages and their application in food safety. Lett. Appl. Microbiol. 47, 479–485. doi: 10.1111/j.1472-765X.2008.02458.x 19120914

[ref19] Gordillo Altamirano F. Forsyth J. H. Patwa R. Kostoulias X. Trim M. Subedi D.. (2021). Bacteriophage-resistant *Acinetobacter baumannii* are resensitized to antimicrobials. Nat. Microbiol. 6, 157–161. doi: 10.1038/s41564-020-00830-7 33432151

[ref20] Guzman L.-M. Belin D. Carson M. J. Beckwith J. (1995). Tight regulation, modulation, and high-level expression by vectors containing the arabinose PBAD promoter. J. Bacteriol. 177, 4121–4130. doi: 10.1128/jb.177.14.4121-4130.1995, PMID: 7608087 PMC177145

[ref21] Hagens S. Loessner M. J. (2007). Application of bacteriophages for detection and control of foodborne pathogens. Appl. Microbiol. Biotechnol. 76, 513–519. doi: 10.1007/s00253-007-1031-8 17554535

[ref22] Harris L. S. Oriel P. J. (1989). “Heteropolysaccharide produced by *Enterobacter sakazakii*” U.S. Patent number 4:636–806.

[ref23] Hayashi K. Morooka N. Yamamoto Y. Fujita K. Isono K. Choi S.. (2006). Highly accurate genome sequences of *Escherichia coli* K-12 strains MG1655 and W3110. Mol. Syst. Biol. 2:0007. doi: 10.1038/msb4100049 PMC1681481 16738553

[ref24] Henderson J. C. O’Brien J. P. Brodbelt J. S. Trent M. S. (2013). Isolation and chemical characterization of lipid a from gram-negative bacteria. J. Vis. Exp. 79:e50623. doi: 10.3791/50623 PMC3885993 24084191

[ref25] Hurrell E. Kucerova E. Loughlin M. Caubilla-Barron J. Forsythe S. J. (2009). Biofilm formation on enteral feeding tubes by *Cronobacter sakazakii*, *Salmonella* serovars and other *Enterobacteriaceae*. Int. J. Food Microbiol. 136, 227–231. doi: 10.1016/j.ijfoodmicro.2009.08.007, PMID: 19720416

[ref26] Iversen C. Lane M. Forsythe S. J. (2004). The growth profile, thermotolerance and biofilm formation of *Enterobacter sakazakii* grown in infant formula milk. Lett. Appl. Microbiol. 38, 378–382. doi: 10.1111/j.1472-765X.2004.01507.x 15059207

[ref27] Jiménez N. Senchenkova S. N. Knirel Y. A. Pieretti G. Corsaro M. M. Aquilini E.. (2012). Effect of LPS biosynthesis mutants on K1 polysaccharide association with *Escherichia coli* cell surface. J. Bacteriol. 194, 3356–3367. doi: 10.1128/jb.00329-12, PMID: 22522903 PMC3434737

[ref28] Kandhai M. C. Reij M. W. Gorris L. G. M. Guillaume-Gentil O. van Schothorst M. (2004). Occurrence of *Enterobacter sakazakii* in food production environments and households. Lancet 363, 39–40. doi: 10.1016/S0140-6736(03)15169-0, PMID: 14723994

[ref29] Kilonzo-Nthenge A. Rotich E. Godwin S. Nahashon S. Chen F. (2012). Prevalence and antimicrobial resistance of *Cronobacter sakazakii* isolated from domestic kitchens in middle Tennessee, United States. J. Food Prot. 75, 1512–1517. doi: 10.4315/0362-028X.JFP-11-442, PMID: 22856579

[ref30] Kim K. Kim K.-P. Choi J. Lim J.-A. Lee J. Hwang S.. (2010). Outer membrane proteins a (OmpA) and X (OmpX) are essential for basolateral invasion of *Cronobacter sakazakii*. Appl. Environ. Microbiol. 76, 5188–5198. doi: 10.1128/AEM.02498-09, PMID: 20543055 PMC2916488

[ref31] Kim K.-P. Klumpp J. Loessner M. J. (2007). *Enterobacter sakazakii* bacteriophages can prevent bacterial growth in reconstituted infant formula. Int. J. Food Microbiol. 115, 195–203. doi: 10.1016/j.ijfoodmicro.2006.10.029 17196280

[ref32] Kim M. Ryu S. (2011). Characterization of a T5-like coliphage, SPC35, and differential development of resistance to SPC35 in *Salmonella enterica* serovar typhimurium and *Escherichia coli*. Appl. Environ. Microbiol. 77, 2042–2050. doi: 10.1128/AEM.02504-10, PMID: 21257810 PMC3067339

[ref33] Kim M. Ryu S. (2012). Spontaneous and transient defence against bacteriophage by phase-variable glucosylation of O-antigen in *Salmonella enterica* serovar typhimurium. Mol. Microbiol. 86, 411–425. doi: 10.1111/j.1365-2958.2012.08202.x, PMID: 22928771

[ref34] Kim S. Yoon H. Ryu S. (2018). New virulence factor CSK29544_02616 as LpxA binding partner in *Cronobacter sakazakii*. Sci. Rep. 8:835. doi: 10.1038/s41598-018-19306-0, PMID: 29339761 PMC5770445

[ref35] Labrie S. J. Samson J. E. Moineau S. (2010). Bacteriophage resistance mechanisms. Nat. Rev. Microbiol. 8, 317–327. doi: 10.1038/nrmicro2315 20348932

[ref36] Lee J.-H. Bai J. Shin H. Kim Y. Park B. Heu S.. (2016). A novel bacteriophage targeting *Cronobacter sakazakii* is a potential biocontrol agent in foods. Appl. Environ. Microbiol. 82, 192–201. doi: 10.1128/AEM.01827-15, PMID: 26497465 PMC4702651

[ref37] Lee J. H. Choi Y. Shin H. Lee J. Ryu S. (2012). Complete genome sequence of *Cronobacter sakazakii* temperate bacteriophage phiES15. J. Virol. 86, 7713–7714. doi: 10.1128/jvi.01042-12, PMID: 22733879 PMC3416292

[ref38] Lehner A. Riedel K. Eberl L. Breeuwer P. Diep B. Stephan R. (2005). Biofilm formation, extracellular polysaccharide production, and cell-to-cell signaling in various strains:: aspects promoting environmental persistence. J. Food Prot. 68, 2287–2294. doi: 10.4315/0362-028x-68.11.2287 16300064

[ref39] Lucas A. Cole T. (1990). Breast milk and neonatal necrotising enterocolitis. Lancet 336, 1519–1523. doi: 10.1016/0140-6736(90)93304-8 1979363

[ref40] Majkowska-Skrobek G. Markwitz P. Sosnowska E. Lood C. Lavigne R. Drulis-Kawa Z. (2021). The evolutionary trade-offs in phage-resistant *Klebsiella pneumoniae* entail cross-phage sensitization and loss of multidrug resistance. Environ. Microbiol. 23, 7723–7740. doi: 10.1111/1462-2920.15476, PMID: 33754440

[ref41] Muytjens H. Zanen H. Sonderkamp H. Kollee L. Wachsmuth I. K. Farmer J. (1983). Analysis of eight cases of neonatal meningitis and sepsis due to *Enterobacter sakazakii*. J. Clin. Microbiol. 18, 115–120. doi: 10.1128/jcm.18.1.115-120.1983, PMID: 6885983 PMC270753

[ref42] Niu T. Guo L. Luo Q. Zhou K. Yu W. Chen Y.. (2020). Wza gene knockout decreases *Acinetobacter baumannii* virulence and affects Wzy-dependent capsular polysaccharide synthesis. Virulence 11, 1–13. doi: 10.1080/21505594.2019.1700659, PMID: 31878839 PMC6961727

[ref43] Pariza M. W. Iandolo J. J. (1974). Determination of genome size of selected typing bacteriophages of *Staphylococcus aureus*. Appl. Environ. Microbiol. 28, 510–512. doi: 10.1128/am.28.3.510-512.1974, PMID: 4278950 PMC186754

[ref44] Pitout J. Moland E. Sanders C. Thomson K. Fitzsimmons S. (1997). Beta-lactamases and detection of beta-lactam resistance in *Enterobacter* spp. Antimicrob Agents Chemother. 41, 35–39. doi: 10.1128/AAC.41.1.35, PMID: 8980751 PMC163656

[ref45] Rakhuba D. Kolomiets E. Szwajcer Dey E. Novik G. (2010). Bacteriophage receptors, mechanisms of phage adsorption and penetration into host cell. Pol. J. Microbiol. 59, 145–155. doi: 10.33073/pjm-2010-023 21033576

[ref46] Regeimbal J. M. Jacobs A. C. Corey B. W. Henry M. S. Thompson M. G. Pavlicek R. L.. (2016). Personalized therapeutic cocktail of wild environmental phages rescues mice from *Acinetobacter baumannii* wound infections. Antimicrob. Agents Chemother. 60, 5806–5816. doi: 10.1128/AAC.02877-15, PMID: 27431214 PMC5038255

[ref47] Reznikoff W. S. Siegele D. A. Cowing D. W. Gross C. A. (1985). The regulation of transcription initiation in bacteria. Annu. Rev. Genet. 19, 355–387. doi: 10.1146/annurev.ge.19.120185.002035 3936407

[ref48] Roach D. R. Leung C. Y. Henry M. Morello E. Singh D. Di Santo J. P.. (2017). Synergy between the host immune system and bacteriophage is essential for successful phage therapy against an acute respiratory pathogen. Cell Host Microbe 22:38-+. doi: 10.1016/j.chom.2017.06.018, PMID: 28704651

[ref49] Saitou N. Nei M. (1987). The neighbor-joining method: a new method for reconstructing phylogenetic trees. Mol. Biol. Evol. 4, 406–425. doi: 10.1093/oxfordjournals.molbev.a040454, PMID: 3447015

[ref50] Salazar K. C. Ma L. Green S. I. Zulk J. J. Trautner B. W. Ramig R. F.. (2021). Antiviral resistance and phage counter adaptation to antibiotic-resistant Extraintestinal pathogenic *Escherichia coli*. MBio 12:211. doi: 10.1128/mBio.00211-21 PMC8092219 33906920

[ref51] Shin H. Lee J.-H. Kim Y. Ryu S. (2012). Complete genome sequence of *Cronobacter sakazakii* bacteriophage CR3. J. Virol. 86, 6367–6368. doi: 10.1128/jvi.00636-12, PMID: 22570242 PMC3372181

[ref52] Sirisena D. M. Brozek K. A. MacLachlan P. R. Sanderson K. E. Raetz C. R. (1992). The rfaC gene of *Salmonella typhimurium*. Cloning, sequencing, and enzymatic function in heptose transfer to lipopolysaccharide. J. Biol. Chem. 267, 18874–18884. doi: 10.1016/S0021-9258(19)37042-5, PMID: 1527014

[ref53] Song L. Yang X. Huang J. Zhu X. Han G. Wan Y.. (2021). Phage selective pressure reduces virulence of Hypervirulent *Klebsiella pneumoniae* through mutation of the wzc gene. Front. Microbiol. 12:739319. doi: 10.3389/fmicb.2021.739319, PMID: 34690983 PMC8526901

[ref54] Stothard P. Wishart D. S. (2005). Circular genome visualization and exploration using CGView. Bioinformatics 21, 537–539. doi: 10.1093/bioinformatics/bti054 15479716

[ref55] Strauch E. Hammerl J. Hertwig S. (2007). Bacteriophages: new tools for safer food? J. Verbr. Lebensm. 2, 138–143. doi: 10.1007/s00003-007-0188-5

[ref56] Sullivan M. J. Petty N. K. Beatson S. A. (2011). Easyfig: a genome comparison visualizer. Bioinformatics 27, 1009–1010. doi: 10.1093/bioinformatics/btr039, PMID: 21278367 PMC3065679

[ref57] Tamura K. Stecher G. Kumar S. (2021). MEGA11: molecular evolutionary genetics analysis version 11. Mol. Biol. Evol. 38, 3022–3027. doi: 10.1093/molbev/msab120, PMID: 33892491 PMC8233496

[ref58] Tang M. Huang Z. Zhang X. Kong J. Zhou B. Han Y.. (2023). Phage resistance formation and fitness costs of hypervirulent *Klebsiella pneumoniae* mediated by K2 capsule-specific phage and the corresponding mechanisms. Front. Microbiol. 14:1156292. doi: 10.3389/fmicb.2023.1156292, PMID: 37538841 PMC10394836

[ref59] Tanji Y. Shimada T. Fukudomi H. Miyanaga K. Nakai Y. Unno H. (2005). Therapeutic use of phage cocktail for controlling *Escherichia coli* O157:H7 in gastrointestinal tract of mice. J. Biosci. Bioeng. 100, 280–287. doi: 10.1263/jbb.100.280 16243277

[ref60] Tanji Y. Shimada T. Yoichi M. Miyanaga K. Hori K. Unno H. (2004). Toward rational control of *Escherichia coli* O157:H7 by a phage cocktail. Appl. Microbiol. Biotechnol. 64, 270–274. doi: 10.1007/s00253-003-1438-9, PMID: 13680205

[ref61] Tompkin R. B. (2002). Microorganisms in foods 7: Microbiological testing in food safety management. New york: Kluwer Academic/Plenum publishiers.

[ref62] Van Acker J. De Smet F. Muyldermans G. Bougatef A. Naessens A. Lauwers S. (2001). Outbreak of necrotizing enterocolitis associated with *Enterobacter sakazakii* in powdered milk formula. J. Clin. Microbiol. 39, 293–297. doi: 10.1128/JCM.39.1.293-297.2001, PMID: 11136786 PMC87717

[ref63] Wang L. Hu X. Tao G. Wang X. (2012). Outer membrane defect and stronger biofilm formation caused by inactivation of a gene encoding for heptosyltransferase I in *Cronobacter sakazakii* ATCC BAA-894. J. Appl. Microbiol. 112, 985–997. doi: 10.1111/j.1365-2672.2012.05263.x, PMID: 22353600

[ref64] Wang X. Loh B. Gordillo Altamirano F. Yu Y. Hua X. Leptihn S. (2021). Colistin-phage combinations decrease antibiotic resistance in *Acinetobacter baumannii* via changes in envelope architecture. Emerg. Microbes Infect. 10, 2205–2219. doi: 10.1080/22221751.2021.2002671, PMID: 34736365 PMC8648044

[ref65] Wang L. Pang X. Zhao J. Jin H. Yang X. Fu S.. (2022). Isolation and characteristics of new phage JK004 and application to control *Cronobacter sakazakii* on material surfaces and powdered infant formula. LWT 153:112571. doi: 10.1016/j.lwt.2021.112571

[ref66] Yang Y. Shen W. Zhong Q. Chen Q. He X. Baker J. L.. (2020). Development of a bacteriophage cocktail to constrain the emergence of phage-resistant *Pseudomonas aeruginosa*. Front. Microbiol. 11:327. doi: 10.3389/fmicb.2020.00327, PMID: 32194532 PMC7065532

[ref67] Zdobnov E. M. Apweiler R. (2001). InterProScan–an integration platform for the signature-recognition methods in InterPro. Bioinformatics 17, 847–848. doi: 10.1093/bioinformatics/17.9.847, PMID: 11590104

[ref68] Zuber S. Boissin-Delaporte C. Michot L. Iversen C. Diep B. Brüssow H.. (2008). Decreasing *Enterobacter sakazakii* (*Cronobacter* spp.) food contamination level with bacteriophages: prospects and problems. Microb. Biotechnol. 1, 532–543. doi: 10.1111/j.1751-7915.2008.00058.x, PMID: 21261874 PMC3815295

