# Supplementary material for: Targeted dual-receptor phage cocktail against Cronobacter sakazakii: insights into phage-host interactions and resistance mechanisms
Source: Front Microbiol. 2024 Dec 6;15:1468686. doi: 10.3389/fmicb.2024.1468686 (PMC11659082; doi:10.3389/fmicb.2024.1468686)
Supplement: Supplementary file 2 [file Presentation_1.PDF]

Supplementary Material

Supplementary Figures 1-3

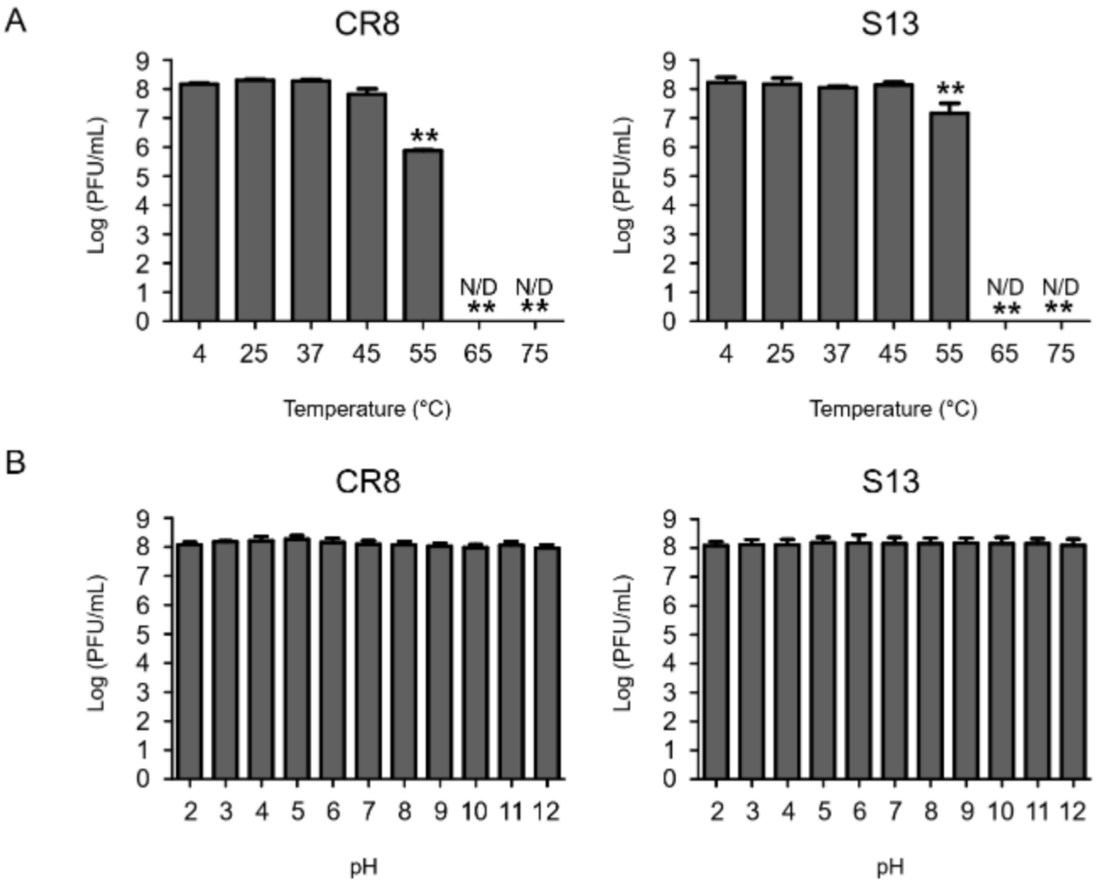

Supplementary Figure 1. Thermal and pH stability of the isolated phages. The viability of *Cronobacter* phages CR8 and S13 were assessed after incubation for 1 h at various ranges of (A) temperatures and (B) pHs. The detection limit was 1 log PFU/ml. Each column displays the Mean ± Standard Deviation (SD) of triplicate experiments. (N/D, not detected). $^{**}P < 0.05$ indicates a significant reduction at temperature 55°C, 65°C, and 75 °C in comparison to initial PFU count.

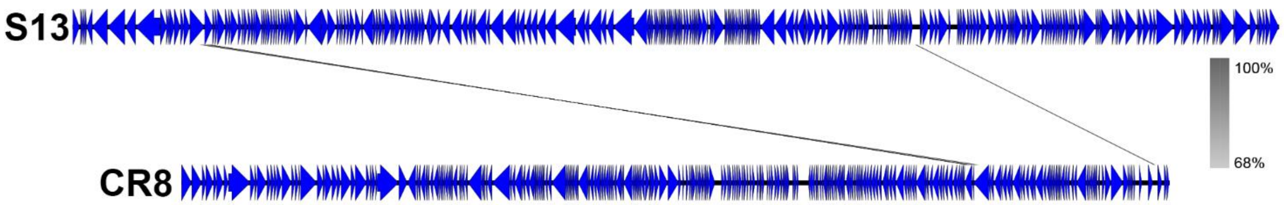

Supplementary Figure 2. Comparative genome analysis between phage S13 and CR8. Each genome was aligned using BLASTn and visualized with Easyfig.

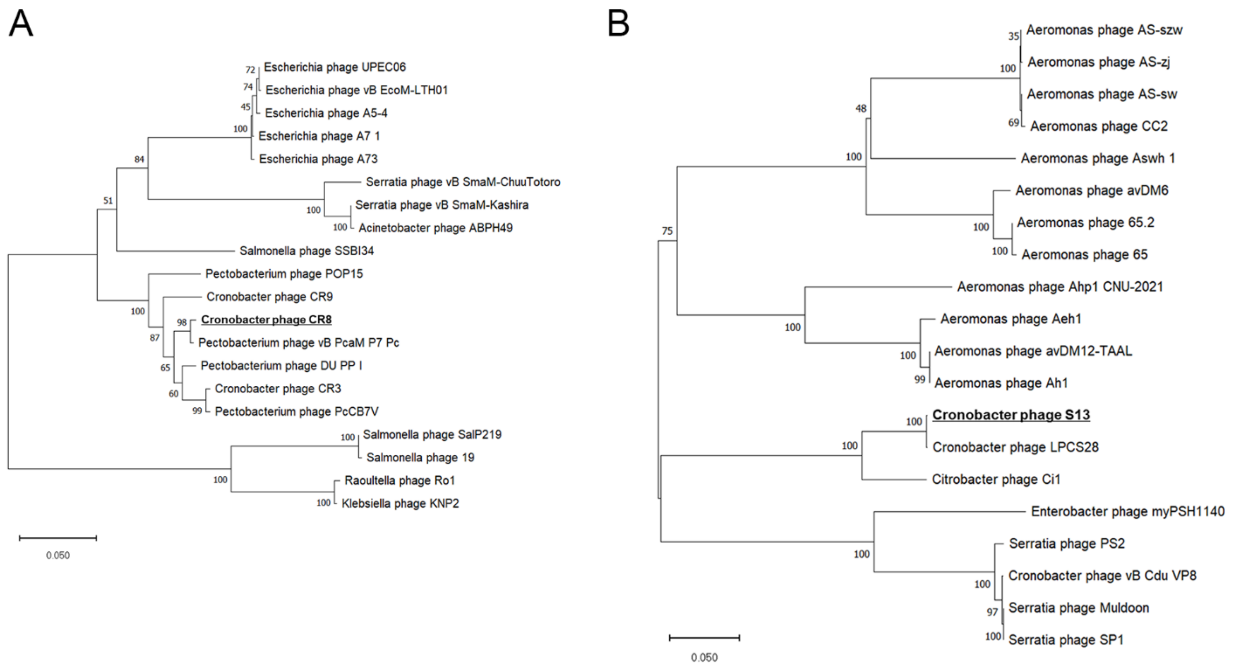

Supplementary Figure 3. Neighbor-joining phylogenetic trees of phage CR8 (A) and phage S13 (B). The amino acid sequences of a terminase large subunit were obtained from the NCBI database and aligned using MUSCLE. The phylogenetic trees were generated with MEGA 11.0.13. The numbers at the branch nodes indicate the bootstrap value (%) built on 1000 replication.
